# HIV continues to spread among men who have sex with men in Georgia; time for action

**DOI:** 10.1371/journal.pone.0214785

**Published:** 2019-04-09

**Authors:** Ali Mirzazadeh, Atefeh Noori, Natia Shengelia, Ivdity Chikovani

**Affiliations:** 1 Department of Epidemiology and Biostatistics, Institute for Global Health Sciences, University of California San Francisco, San Francisco, California, United States of America; 2 HIV/STI Surveillance Research Center, and WHO Collaborating Center for HIV Surveillance, Institute for Futures Studies in Health, Kerman University of Medical Sciences, Kerman, Iran; 3 Department of Health Research Methods, Evidence, and Impact (HEI), McMaster University, Ontario, Hamilton, Canada; 4 Curatio International Foundation, Tbilisi, Georgia; National and Kapodistrian University of Athens, GREECE

## Abstract

**Introduction:**

In order to determine the impact of HIV prevention and care programs, it is essential to look at both HIV incidence and prevalence estimates and trends over time. We estimated the HIV incidence and prevalence and assessed the trend using data from three cross-sectional surveys of men who have sex with men (MSM) in two cities in Georgia.

**Methods:**

Using respondent-driven sampling strategy, a total of 796 eligible MSM (18 years or older men with self-reported oral or anal sex with another man in past 12 months) were recruited in Tbilisi in 2010, 2012 and 2015 and 115 in Batumi 2015 into behavioral surveys and HIV testing. To estimate the HIV incidence, we divided the number MSM tested positive for HIV to the time at risk. We calculated the time at risk as years since age at first anal intercourse to the age at last HIV-negative test or the age at first HIV-positive test, accounted for the interval censorship. We calculated the respondent-driven sampling adjusted estimates for HIV prevalence and assessed the trend in Tbilisi by Chi2 test for trend. For HIV incidence rate, we used Kaplan Meier method to estimate the rates and assessed the subgroup differences by log-rank test.

**Results:**

The HIV prevalence was 14.9% in Batumi in 2015; it significantly increased in Tbilisi from 6.2% in 2010 to 14.1% in 2012, and to 19.6% in 2015 (p-value for trend < 0.001). Likewise, the HIV incidence rate in Tbilisi significantly increased form 0.45 per 100 person-years (PY) in 2010 to 0.98 per 100 PY in 2012 (p-value 0.01), and to 1.63 per 100 PY in 2015 (p-value < 0.001). HIV incidence rate was 1.37 per 100 PY in Batumi in 2015. In 2015, young MSM (Tbilisi: 3.71, Batumi: 3.92 per 100 PY, p-value< 0.008), single MSM (Tbilisi: 1.99, per 100 PY, p-value 0.03) and less educated MSM (Batumi: 1.86 per 100 PY, p-value 0.03) had higher HIV incidence than other MSM.

**Conclusion:**

Our findings suggest the continuous transmission of HIV among MSM in Tbilisi and a high prevalence of HIV among MSM in Batumi and the critical need for scaling up the coverage and accessibility of combination prevention packages including rapid HIV diagnosis and treatment.

## Introduction

Eastern Europe and Central Asia (EECA) is the home for 1.6 million people living with HIV. It is the only region in the world where the HIV epidemic continues to grow; 57% increase in annual new HIV infections between 2010 and 2015 [1]. HIV epidemic in Georgia, a country in EECA region, is concentrated among men who have sex with men (MSM) and people who inject drugs [2] Up to mid-February 2018, 6,882 (5,153 men) cases of HIV have been registered; the majority were 29–40 years old. Since 2016, the annual number of HIV registered cases have been increased continuously [3]. Injecting drug use was a primary HIV transmission route until 2012 [2], after which sexual transmission became the leading route, reaching 51.5% of all cases in 2016 [4]. Number of cases most likely acquired via MSM-transmission also increased over time, now accounting for 16.8% of all cases identified in 2016 [4].

We estimate about 17,200 MSM live in Georgia [5]. Finding from MSM survey 2015 showed a high prevalence of unsafe sex among this population which did not improved since 2010. On average, MSM reported having 6 sexual partners per year. In 2015, condom use at last anal intercourse was reported by only 69.6% of MSM participants, while consistent condom use in all sexes in pat month was much less, around 30%. Proportion of MSM who were tested during the last 12 months and received their results increased from 15.8% in 2010 to 38.4% in 2015 in Tbilisi [6], however still majority of MSM are not tested recently for HIV.

The Georgina HIV program provides services to MSM throughout the country via 11 centers (of which 3 located in Tbilisi, 3 in Batumi, 3 in Kutaisi, 1 in Zugdidi and 1 in Telavi). These services include condom and lubricant distribution, HIV and STI testing and counseling either on-site and also via outreach team at MSM hot spots. Yet the coverage of such services is very low; only 3,846 MSM (less than 23% of all MSM) received services in 2017. Since 2017, pilot pre-exposure prophylaxis (PrEP) program was started for 100 MSM in Tbilisi with a plan to expand it to other regions of the country. [7].

To reliably assess the impact of the national response to HIV epidemic in MSM, it is crucial to monitor both HIV prevalence and incidence. Studies to measure HIV incidence require years of follow-up of a large group of negative MSM, which are expensive and most of the time not feasible. Using laboratory assays (that can measure biomarkers of recent infection) to measure HIV incidence are also not reliable in settings where HIV epidemic is only concentrated among key populations [2]. Thus, in this study we use a method proposed by Osmond et al. [8] to estimate the HIV incidence. This study aimed to estimate the prevalence and trend of HIV prevalence and incidence among MSM population in Georgia from 2010 to 2015. We also reported the HIV prevalence and incidence in subgroups of MSM defined by demographic and risk behavior characteristics are presented.

## Material and methods

### Surveys and study population

Three Integrated Bio-Behavioral Surveys (IBBS) were carried out in Georgia in 2010, 2012 and 2015 among MSM using respondent-driven sampling as recruitment strategy. The first (Year: 2010; N = 278) and second (Year: 2012; N = 218) rounds were conducted only in Tbilisi and the third round was conducted in Tbilisi (Year: 2015; N_1_ = 300) and Batumi (Year: 2015; N_2_ = 115) [9][10][6]. The eligibility criteria were the same in all surveys: male aged 18 years and above, had oral or anal sex with men within the past 12 months, resided in Tbilisi or Batumi, ability to understand and communicate in Georgian language and provided informed consent [9,10,6].

Recruitment was started by several seeds (7 in 2010, 6 in 2013, and 9 in 2015) in Tbilisi and (8 in 2015) Batumi. The inclusion criteria for seed selection in all surveys was the same and similar to eligibility criteria for other survey participants (see above). We purposefully selected seeds from different subpopulations of MSM to represent the demographic profile and socially diverse MSM network (age, socio-economic status, occupation, education). After completing the survey, each seed was given three recruitment coupons and trained to use them to invite three eligible MSM peers to the study. Each coupon had a serial number, study location, information about the monetary incentive and a two weeks expiration date. MSM who referred to the study site with a valid recruitment coupon, first assessed for eligibility, and after providing the informed consent, were interviewed by trained interviewers. This process of peer referral was continued to reach the study sample size. Each participant was given a monetary incentive of 15 GEL (~9 USD) in 2010, 10 GEL (~6 USD) in 2013 and 25 GEL (~11 USD) in 2015 to participate in the survey. Additional incentive of 5 GEL (that was ~3 USD in 2010 and 2013 and ~2.25 USD in 2015) for every successful peer referral to the study [9,10,6].

Trained interviewers conducted face to face interview using an interviewer-administered electronic questionnaire. Following the completion of the behavioral questionnaire, participants were asked to voluntarily provide blood sample for HIV test. More than 97% of study population were agreed and provided blood sample in each survey. All blood samples were tested for HIV-Ab using an enzyme-linked immunosorbent assay (ELISA), and if positive, were confirmed by Western Blot HIV Blot 2.2, MP Biomedical on the same blood sample. All HIV tests were done at the Infectious Diseases, AIDS and Clinical Immunology Research Center laboratory [9,10,6].

### Subpopulations and main sexual behaviours covariates

Our standardized questionnaire for all surveys included sections for several questions on socio-demographic characteristics, drug use, sexual behavior including engagement in commercial sex, condoms and lubricants use, HIV knowledge, and use of prevention program including HIV testing. The survey questionnaires in both English and Georgian languages are accessible through ‘Supporting Information’. In this paper, we analyzed the data for different subgroups of MSM as defined by age (≤24, >24), education years (≤ 6, > 6 years), Marital status (Married, Single), drug use in past 12 months (no drug use, only non-injecting, injecting), and condom use in last intercourse with different types of partners (yes, no). Participants were asked about sexual relationship with different types of partners including occasional, commercial, and regular partners. An occasional partner was defined as a sex partner with whom sexual contact was established without exchange for material remuneration, for a short period of time, who is not a spouse, a regular partner, or a sex worker. A commercial partner was defined as a sex partner with whom sexual contact was established in exchange for material remuneration, meaning that they paid money or gave some other material remuneration to the partner. A regular sex partner was defined as when repeatedly uses sexual services of a particular person.

### Defining time at risk and incidence rates

We calculated HIV incidence using the method suggested by Osmond et al. [8]. In this method, the number of HIV positive cases identified in each survey’s round was considered as the numerator. The denominator, i.e. the person-years at risk for HIV were calculated by subtracting the age at the time of interview (for HIV-negative cases) or age at the time of first HIV positive test result (for HIV-positive cases) from the Age at First Anal Intercourse (AFAI).

$$HIV\;incidence\;rate\;=\;\frac{Number\;of\;HIV\;positive\;cases\;}{Current\;age-Age\;at\;first\;anal\;intercourse\;}\;\times \;100$$

The AFAI was not directly measured in MSM surveys. We imputed the AFAI using three methods. In method one, based on the literature review, [11–14] we assumed that all participants had their first anal sex at age of 17 years old (i.e. AFAI = 17). In method two, using data from a cohort study of MSM in Australia, [13] we grouped participants into six age groups. Then, we assigned AFAI 16 to age group 18–20, AFAI 18 to age group 21–25, AFAI 20 to age group 26–35, AFAI 21 to age group 36–45, AFAI 22 to age group 46–55 and AFAI 35 to age group 56 and older. In method three, we assigned a random AFAI to all participants that was between age of 15 to 25 using a uniform distribution; The assigned age has to at least one-year less than the age of participant at the time of survey. The incidence was estimated by using all three methods. Since the trend results were similar, we presented findings from method one. In S1 Table, we presented the trend (sensitivity) analysis for method two and three.

### Statistical analysis

We calculated HIV incidence rates (per 100 person-year) point estimates and 95% confidence intervals (95% CI) using Kaplan Meier method and then compared the results across the three survey rounds by log-rank test. Because a few records were missed for HIV test results, we calculated HIV prevalence only for MSM who had tested for HIV and their test results were available to us. To account for the effect of respondent-driven sampling (RDS) strategy, we used Gile’s SS method to calculate the RDS-adjusted estimates using R [15]. All other analyses were done in STATA v.12. The study protocols and questionnaires were approved by the National Ethical Committee of the HIV/AIDS Patients Support Foundation (certificates: #518/619 of 20.08.2010; # 623/724 of 28.06.2012; # 776/877 of 30.01.2015).

## Results

### Demographic and risk behaviors

About one-third of participants were 24 years old or younger; the median age was 27 to 29 years (Table 1). In 2015, more participants were single (89.6% in Tbilisi), had Georgian nationally (99.0% in Tbilisi), and less educated (54.0% in Tbilisi) than those participated in previous surveys. Notably only 2.3% of MSM in Tbilisi and 2.6% of MSM in Batumi reported drug injection during the last year in 2015. The prevalence of non-injecting and injecting drug use, condom use with male, regular or occasional male partner, number of sex with female partners and condom use with female partners was stable overtime in Tbilisi. More participants reported condom use at last sex with commercial male partner in recent years in Tbilisi (79.5% in 2015, p-value for trend 0.02). The median number of sexual partners in last 12 months increased from 4 to 6 persons in 2015 in Tbilisi, while it was 2 persons in Batumi. More participants reported recent HIV tests in survey 2015 in Tbilisi (70.6%, p-value for trend <0.001) and Batumi (79.7%).

**Table 1:** Demographic and behavioral characteristics of participants in three repeated cross-sectional surveys of men who have sex with men; Georgia, 2010–2015.

Characteristic	Tbilisi				Batumi
	2010	2012	2015	P-value for trend	2015
	n (%) (N = 278)	n (%) (N = 218)	n (%) (N = 300)		n(%) (N = 115)
**Demographic**					
Age (years)					
≤ 24	86(30.9)	83(38.1)	111(37.0)	0.01	35(30.4)
> 24	192 (69.1)	135 (61.9)	189 (63.0)		80(69.5)
Median age (years)	29.0	27.0	28.0	----	29
Education years					
No education or ≤ 6	153 (55.0)	98 (45.0)	162 (54.0)	0.05	67(58.2)
More than 6	125 (45.0)	120 (55.0)	138 (46.0)		48(41.7)
Georgian nationality	228 (82.0)	196 (89.9)	297 (99.0)	<0.001	113(98.2)
Marital status					
Married	54 (19.5)	30 (13.8)	31 (10.3)	0.007	22(19.1)
Single	223(80.5)	188(86.2)	269(89.6)		93(80.8)
**Drug use history during the last 12 months**					
No drug use or injection	212(76.2)	177(81.1)	241 (80.3)	0.59	69(60)
Non-injecting drug use	54 (19.4)	35 (16.0)	52 (17.3)		43(37.3)
Injecting drug use	12 (4.3)	6 (2.7)	7 (2.3)		3(2.6)
**Sexual behavior**					
Median # of anal/oral partners in the last 12 months	4.0	4.0	6.0	----	2
Used condom at last anal intercourse with a male partner^1^	181 (68.6)	153 (73.2)	177 (65.0)	0.11	95(83.3)
Used condom at last anal intercourse with a male regular partner^1^	117(64.6)	102(68.5)	127(59.6)	0.21	71(68.2)
Used condom at last anal intercourse with a male commercial partner^1^	48 (64.0)	18 (81.8)	39 (79.5)	0.02	14(93.3)
Used condom at last anal intercourse with a male occasional partner^1^	141(70.1)	97(75.8)	143(69.0)	0.39	72(83.7)
Had female partner in the last 12 months	173 (71.4)	112 (68.7)	168 (78.5)	0.07	60(69.7)
Used condom at last intercourse with a female partner^1^	100 (57.8)	77 (68.8)	112 (67.4)	0.14	42(70)
**HIV testing history**					
Tested for HIV in the last year	73 (61.8)	74 (55.6)	125 (70.6)	<0.001	59(79.7)
Tested for HIV between 1 and 2 years ago	14(11.8)	33(24.8)	27(15.2)		8(10.8)
Tested for HIV more than 2 years ago	31(26.2)	26(19.5)	25(14.1)		7(9.4)

^1^ Only among participants who reported having sex with such kind of persons in the last 12 months.

### HIV prevalence

**Fig 1:**
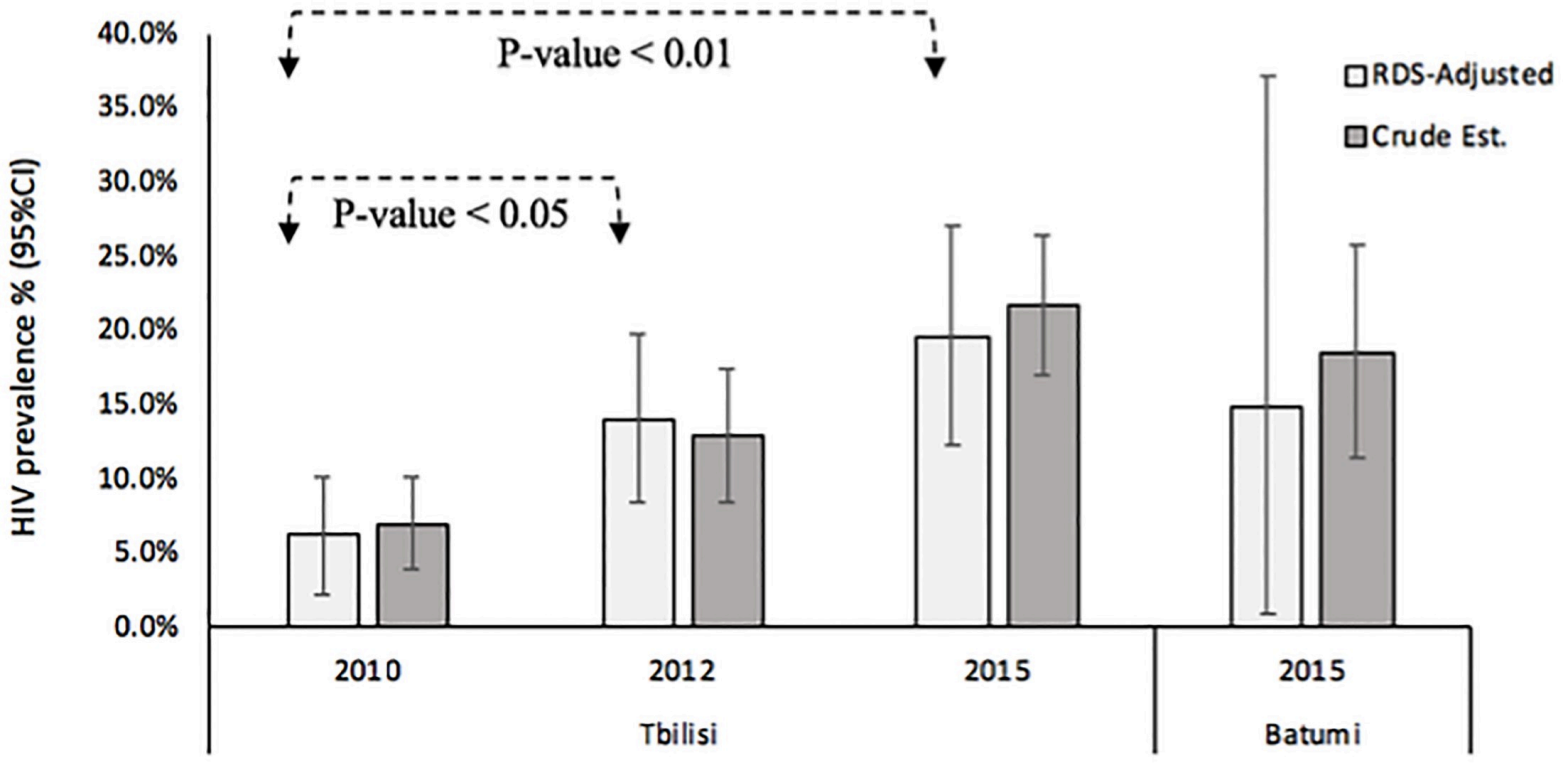
Trend in HIV prevalence among men who have sex with men in Georgia, 2010 to 2015. Gile’s SS method was used to calculate the RDS-adjusted estimates. Crude estimates were calculated assuming simple random sampling as the recruitment strategy.

In Tbilisi, the RDS-adjusted HIV prevalence significantly increased (p-value for trend <0.001) form 6.2% in 2010 to 14.1% in 2012, and to 19.6% in 2015 (Fig 1). In Tbilisi (Table 2), HIV prevalence increased significantly since 2010 in both younger (p-value = 0.001) and older MSM (p-value <0.001), in those with less education (p-value <0.001), single MSM (p-value <0.001), those with no history of drug use/injection (p-value <0.001) and those who reported non-injecting drug use (p-value = 0.01). The HIV prevalence also increased significantly almost in all MSM subgroups regardless of their type of partners and condom use behaviors in Tbilisi. In survey 2015 in Tbilisi, HIV prevalence was higher among MSM with lower level of education (25.3% vs. 17.3%, p-value 0.01). The overall HIV prevalence among MSM in Batumi was 14.9% in 2015 (Fig 1); it was significantly higher among MSM with lower level of education (24.6 vs. 10.4%, p-value 0.01) (Table 2).

**Table 2:** HIV prevalence by demographic and behavioral characteristics of participants in three repeated cross-sectional surveys of men who have sex with men; Georgia, 2010–2015.

Characteristic	Tbilisi							Batumi	
	2010		2012		2015		Trend P-value	2015	
	n/N (%)	p-value	n/N (%)	p-value	n/N (%)	p-value		n/N (%)	p-value
**Demographic**									
Age, year									
≤ 24	3/83(3.6)	0.14	4/81(4.9)	0.006	20/111(18.0)	0.26	0.001	6/35(17.1)	0.79
> 24	16/188(8.5)		24/134(17.9)		45/189(23.8)		<0.001	15/78(19.2)	
Education year									
No education or ≤ 6	10/148(6.8)	0.85	12/98(12.4)	0.75	41/162(25.3)	0.01	<0.001	16/65(24.6)	0.05
More than 6	9/123(7.3)		16/117(13.7)		24/138(17.3)		0.05	5/48(10.4)	
Current marital status									
Married	5/53(9.4)	0.44	4/30(13.3)	0.95	6/31(19.3)	0.50	0.43	3/21(14.2)	0.5
Single	14/217(6.4)		24/185(12.9)		59/269(21.9)		<0.001	18/92(19.5)	
**Drug use history during the last 12 months**									
No drug use or injection	18/208(8.6)	0.15	23/174 (13.2)	0.92	55/241(22.8)	0.05	<0.001	17/67(25.3)	0.07
Non-injecting drug use	0/12(0)		4/35(11.4)		10/52(19.2)		0.01	4/43(9.3)	
Injecting drug use	1/51(1.9)		1/6(16.6)		0/10(0)		0.19	0/3(0)	
**Sexual behaviors**									
Used condom at last anal intercourse with a male partner^1^									
Yes	14/176(7.9)	0.20	17/151(11.2)	0.16	42/177(23.7)	0.83	<0.001	17/93(18.2)	0.77
No	3/81(3.7)		10/53(18.87)		22/95(23.1)		0.001	4/19(21.0)	
Used condom at last anal intercourse with a male regular partner^1^									
Yes	9/114(7.9)	0.42	12/100(12.0)	0.62	29/127(22.8)	0.34	0.003	12/70(7.1)	0.84
No	3/63(4.7)		7/47(14.89)		24/86(27.9)		0.001	6/32(18.7)	
Used condom at last anal intercourse with a male commercial partner^1^									
Yes	2/47(4.3)	0.90	2/18(11.1)	0.54	13/39(33.3)	0.28	<0.001	5/13(38.4)	0.43
No	1/27(3.7)		0/3(0)		2/10(20)		0.21	0/1(0)	
Used condom at last anal intercourse with a male occasional partner^1^									
Yes	11/137(8.0)	0.76	11/96(11.4)	0.23	33/143(23.0)	0.90	0.001	12/70(17.1)	0.70
No	4/59(6.7)		6/30(20)		14/64(21.8)		0.05	3/14(21.4)	
Had female partner in the last 12 months									
Yes	10/168(5.9)	0.66	12/109(11.0)	0.88	32/168(19.0)	0.07	0.001	9/58(15.5)	0.21
No	5/67(7.4)		6/51(11.7)		13/46(28.2)		0.007	7/26(26.9)	
Used condom at last intercourse with a female partner^1^									
Yes	7/99(7.1)	0.50	8/75(10.6)	0.86	26/112(23.2)	0.29	0.002	5/42(11.9)	0.21
No	3/66(4.5)		4/34(11.7)		6/54(11.1)		0.32	4/16(25)	

^1^Only among participants who reported having sex with such kind of persons in the last 12 months.

There are some missing values regarding the HIV prevalence. (Table reflects calculation for only MSM who were tested on HIV during the survey)

### HIV incidence

**Fig 2:**
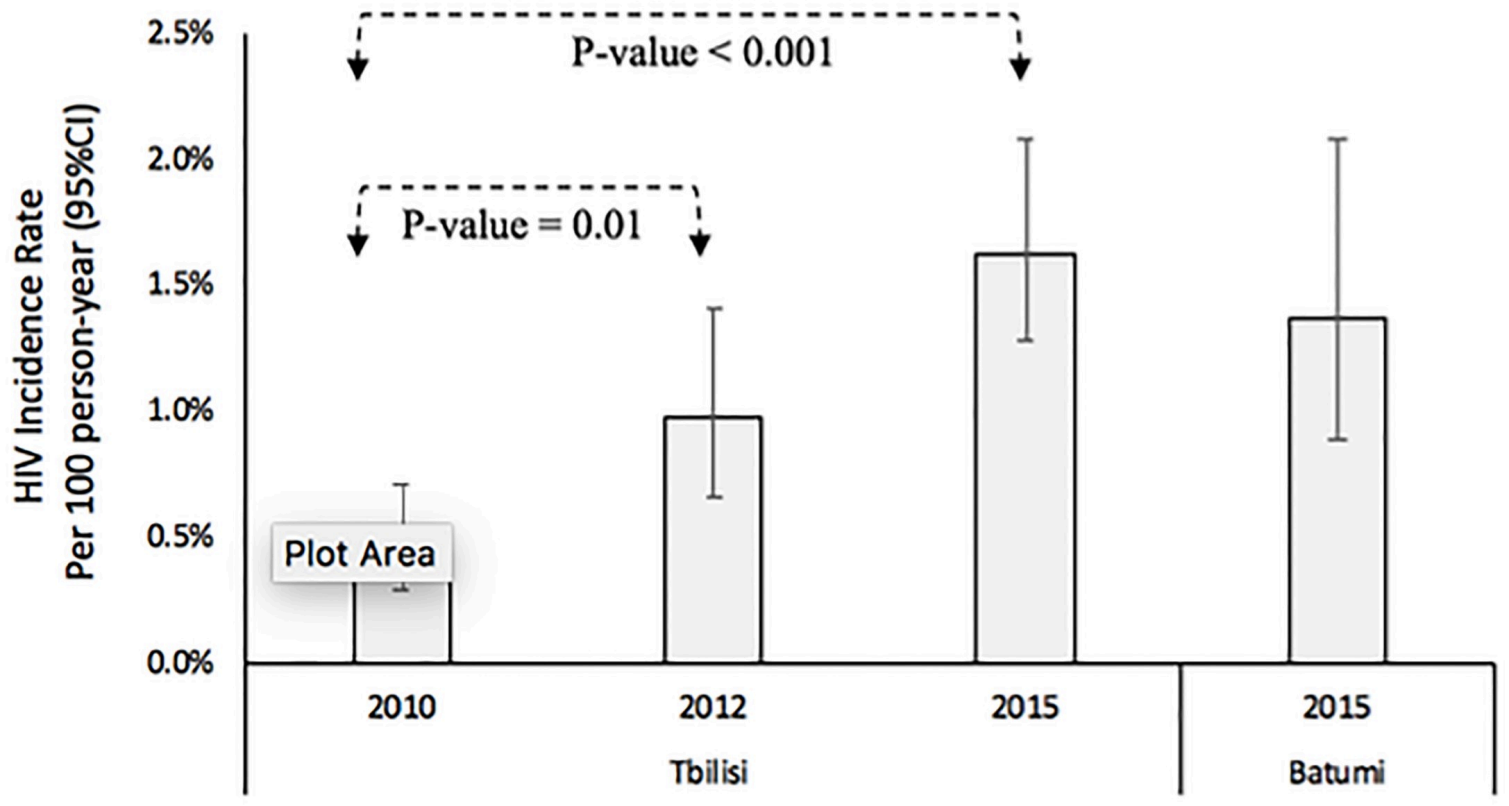
Trend in HIV incidence rate per 100 person-years among men who have sex with men in Georgia, 2010 to 2015.

In Tbilisi, the HIV incidence rate (Fig 2) significantly increased from 0.45 per 100 person-years in 2010 to 0.98 per 100 person-years in 2012 (p-value = 0.01), and to 1.63 per 100 person-years in 2015 (p-value <0.001). In Tbilisi (Table 3), HIV incidence rate significantly increased since 2010 in both younger (p-value < 0.001) and older MSM (p-value <0.001), in those with less education (p-value <0.001), single MSM (p-value <0.001), those with no history of drug use/injection (p-value <0.001) and those who reported non-injecting drug use (p-value < 0.001). The HIV incidence rate was also increased significantly almost in all MSM subgroups regardless of their type of partners and condom use behaviors in Tbilisi. In survey 2015 in Tbilisi, younger MSM (3.71 per 100 person-years, p-value<0.001), single MSM (1.99, p-value 0.03) and those reported condom use in sex with last female partner (1.84 per 100 person-years, p-value 0.03) had a higher risk for HIV. In Batumi, the overall HIV incidence rate was 1.37 per 100 person-years (Fig 1), which was significantly higher among younger (p-value 0.008), and less educated MSM (p-value 0.03).

**Table 3:** HIV incidence rates by age and some main characteristics in three repeated cross-sectional surveys of men who have sex with men; Georgia, 2010–2015^1^.

Characteristic	Tbilisi										Batumi		
	2010			2012			2015			p-value trend^3^	2015		
	Cases /100 PY	IR (95%CI)^2^	P-value	Cases /100 PY	IR (95% CI)	P-value	Cases /100 PY	IR (95% CI)	P-value		Cases /100 PY	IR (95% CI)	P-value
**Demographic**													
Age in years													
≤ 24	3/425	0.70 (0.22, 2.18)	0.51	4/402	0.99 (0.37, 2.65)	0.95	20/539	3.71 (2.39, 5.75)	<0.001	<0.001	6/153	3.92 (1.76, 8.72)	0.008
> 24	16/3384	0.47 (0.28, 0.77)		24/2257	1.06 (0.71, 1.58)		45/3122	1.44 (1.07, 1.93)		<0.001	15/1385	1.08 (0.65, 1.79)	
Current marital status													
Single	14/2815	0.49 (0.29, 0.83)	0.94	24/2001	1.19 (0.80, 1.78)	0.20	59/2958	1.99 (1.54, 2.57)	0.03	<0.001	18/1146	1.57 (0.98, 2.49)	0.12
Married	5/987	0.50 (0.21, 1.21)		4/2116	0.60 (0.22, 1.61)		6/703	0.85 (0.38, 1.89)		0.69	3/392	0.76 (0.24, 2.37)	
Education, years													
No education or ≤ 6	10/1971	0.50 (0.27, 0.94)	0.94	12/1124	1.06 (0.60, 1.87)	0.94	41/1905	2.15 (1.58, 2.92)	0.07	<0.001	16/859	1.86 (1.14, 3.04)	0.03
More than 6	9/1838	0.48 (0.25, 0.94)		16/1535	1.04 (0.63, 1.70)		24/1756	1.36 (0.91, 2.03)		0.023	5/679	0.73 (0.30, 1.76)	
**Drug use history during the last 12 months**													
No drug use or injection	18/3005	0.59 (0.37, 0.95)	0.32	23/2195	1.04 (0.69, 1.57)	0.85	55/3122	1.76 (1.35, 2.29)	0.21	<0.001	17/956	1.77 (1.10, 2.86)	0.07
Non-injecting drug use	1/629	0.15 (0.02, 1.12)		4/345	1.15 (0.43, 3.08)		10/428	1.47 (1.25, 4.3)		<0.001	4/526	0.76 (0.28, 2.04)	
Injecting drug use	0/175	0		1/119	0.84 (0.11, 5.96)		0/111	0		0.74	0/61	0	
**Sexual behaviors**													
Used condom at last anal intercourse with a male partner^1^													
Yes	14/2355	0.59 (0.35–1.00)	0.15	12/1184	1.01 (0.57–1.78)	0.28	42/2196	1.91 (1.41–2.58)	0.80	<0.001	17/1221	1.39 (0.86, 2.23)	0.47
No	3/ 1231	0.24 (0.07–0.75)		7/ 478	1.46 (0.69–3.07)		22/1081	2.03 (1.34–3.09)		<0.001	4/307	1.30 (0.48, 3.47)	
Used condom at last anal intercourse with a male regular partner													
Yes	9/1492	0.60 (0.31–1.15)	0.51	12/1184	1.01 (0.57–1.78)	0.44	29/1574	1.84 (1.28–2.65)	0.33	0.002	12/901	1.33 (0.75, 2.34)	0.43
No	3/ 790	0.37 (0.12–1.17)		7/478	1.46 (0.69–3.07)		24/ 998	2.40 (1.61–3.58)		0.006	6/499	1.20 (0.54, 2.67)	
Used condom at last anal intercourse with a male commercial partner													
Yes	2/616	0.26 (0.03–1.88)	0.91	2/140	1.42 (0.35–5.71)	0.52	13/452	2.87 (1.67–4.95)	0.75	<0.001	5/190	2.63 (1.09, 6.32)	0.41
No	1/ 376	0.32 (0.03–1.7)		0/54	0		2/ 94	2.12 (0.53–8.50)		0.09	0/7	0	
Used condom at last anal intercourse with a male occasional partner													
Yes	11/1978	0.56 (0.31–1)	0.61	11/1134	0.97 (0.54–1.75)	0.47	33/2844	1.58 (1.18–2.12)	0.74	0.001	12/953	1.25 (0.71, 2.21)	0.31
No	4/1010	0.40 (0.15–1.06)		6/431	1.39 (0.63–3.10)		14/888	1.91 (1.19–3.08)		0.01	3/178	1.68 (0.54, 5.22)	
Had female partner in the last 12 months													
Yes	10/2436	0.41 (0.22–0.76)	0.81	12/1505	0.79 (0.45–1.40)	0.88	32/3251	1.26 (0.93–1.71)	0.17	0.0010	9/777	1.15 (0.60, 2.22)	0.13
No	5/1086	0.46 (0.19–1.10)		6/709	0.84 (0.38–1.88)		13/989	2.02 (1.30–3.13)		0.002	7/339	2.06 (0.98, 4.33)	
Used condom at last intercourse with a female partner^1^													
Yes	7/1358	0.51 (0.24–1.08)	0.41	8/939	0.85 (0.42–1.70)	0.78	26/1412	1.84 (1.25–2.70)	0.03	0.002	5/467	1.07 (0.44, 2.57)	0.38
No	3/1051	0.28 (0.09–0.8)		4/566	0.70 (0.26–1.88)		6/815	0.73 (0.33–1.63)		0.39	4/310	1.29 (0.48, 3.43)	

^1^ According to the first method (17 years old as AFAI);

^2^ Incidence Rate (IR) and 95% Confidence Interval (CI);

^3^ the first p-value estimated for each variable shows test for overall trend

## Discussion

Our findings indicated both prevalence and incidence of HIV have been increased since 2010 among men who have sex with men in Tbilisi, as well as a high HIV prevalence among MSM in Batumi in 2015. About one in five MSM in Georgia is infected with HIV and the rate of HIV infection is estimated as high as 1.63%.

Estimating HIV incidence is important to assess the effectiveness of prevention program by looking at the transmission rate of HIV. Using assay-derived recent infection testing algorithm over the period of 2010–12 on 1155 HIV cases at a referral center in Georgia, more than 20% were determined to be recent infections and the HIV incidence was estimated to be 0.10% to 0.12% [16]. We observed a much higher, 1.56% incidence rate among MSM, 15 times higher than the previously reported incidence. Such high incidence among MSM is concerning and needs to be addressed by Georgian HIV program.

Epidemic of HIV among MSM is continues to expand in Tbilisi. In five years, the HIV prevalence increased from 6.2% to 19.6% and the HIV incidence increased from 0.45 to 1.63%. Such increases in the HIV prevalence and incidence remained the same and significant even after we adjusted the estimates for the effect of seed selection and RDS methodology. Continued expansion of HIV epidemics among MSM was also reported in other countries [17,18]. Among MSM, HIV incidence was reported as 2.0% in England [19], 5.9% in Thailand [20], 6.7% in China [21], and a range of 0.70 to 15.0% in Asia [20]. Similar to our findings, the highest increase in new HIV infection is happening among young MSM aged 15–24 years [22].

While not statistically significant due to small sample size, we found in all three surveys in Tbilisi and the one in Batumi, MSM who reported sex with a female partner in last 12 months had a lower prevalence and risk of HIV infection than other MSM who exclusively had sex with men. Such MSM are likely to have less male partners, use condom more frequently, less likely to have receptive anal sex [23]; they also might have more social support, less stigmatized for their MSM behaviors under the cover of marriage or heterosexual sex, [24] and may have better access to HIV prevention services.

We found that MSM who had a history of drug use or injection had less risk of HIV infection than other MSM. While this is not what we expected, it could be due to different drug use or injecting network that MSM who use/inject drug might be connected to. It is also likely that MSM who use/inject drugs have reached by current harm reduction programs in Georgia [25] than those MSM who do not use or inject drugs. This calls for expanding MSM-oriented services in the country as well as need for more research to understand MSM networks and links to other key population including people who use/inject drugs to inform both case findings and targeted interventions.

In Georgia, several bio-medical, structural and behavioral preventive interventions targeted high-risk populations including MSM are in place. Free condom and lubricant are being delivered by preventive centers and outreach activities, early HIV diagnosis is being promoted by providing free HIV testing and counselling throughout the country, among recent initiatives are PrEP. Surveys and program data show some improvements, however still unsafe sex is common and the coverage of prevention programs is extremely low [6]. Strategies to increase access to and use of current prevention services, as well as using innovative strategies is needed to truly change the trajectory of epidemic among MSM in Georgia.

Our findings have five major limitations. First, we estimated the HIV incidence using data from cross-sectional surveys of MSM. Second, we imputed the age at sex debut; however, in the sensitivity analysis we showed that it only changed our incidence estimates slightly and not affected the incidence trend analysis. Third, we assumed people remained at risk for HIV when they became sexually active; we did not exclude periods of stopping sexual activity or practicing safe. Fourth, our study was done only among MSM in two cities in Georgia and the findings may not be generalized to all regions and MSM subgroups in the country. Last, we were not able to measure the refusal rat for participation (i.e. proportion of those who were invited by a peer but did not participate in the study) and its effect on the study outcomes.

With acknowledgment of limitations, our findings suggest the continuous and high transmission of HIV among men who have sex in Tbilisi and a high prevalence of HIV among MSM in Batumi and the need for scaling up the coverage and accessibility of combination prevention packages including rapid HIV diagnosis and treatment.

## Supporting information

S1 Table (DOCX)
