# Supplementary material for: HIV continues to spread among men who have sex with men in Georgia; time for action
Source: PLoS One. 2019 Apr 9;14(4):e0214785. doi: 10.1371/journal.pone.0214785 (PMC6456173; doi:10.1371/journal.pone.0214785)
Supplement: S1 Table — (DOCX) [file pone.0214785.s001.docx]

Supplement Table 1 – Trend in HIV incidence by three methods to impute sex at first sex among men who have sex with men in Georgia, 2010 to 2015

| **Year** | **Cases/100 PY** | **Incidence rate (95% CI)** | **IRR* (95% CI)** | | **P-value (IRR)** | **P-value trend** |
| --- | --- | --- | --- | --- | --- | --- |
| Age 17 years old at first anal intercourse^2^ | | | | | | |
| Survey 2010 - Tbilisi | 19/4193 | 0.45 (0.29-0.71) | 1 | | - | <0.001 |
| Survey 2012 - Tbilisi | 28/2870 | 0.98 (0.67-1.41) | 2.13 (1.19-3.81) | | 0.01 |  |
| Survey 2015 - Tbilisi | 65/3978 | 1.63 (1.28-2.08) | 3.40 (2.07-5.59) | | <0.001 |  |
| Survey 2015 - Batumi | 21/1538 | 1.37 (0.89-2.09) | - | |  |  |
| Age between 16 to 35 years old at first anal intercourse^3^ | | | | | | |
| Survey 2010 - Tbilisi | 19/3809 | 0.57 (0.32-0.78) | 1 | | - | <0.001 |
| Survey 2012 - Tbilisi | 28/2659 | 1.05 (0.73-1.53) | 2.09 (1.16-3.74) | | 0.01 |  |
| Survey 2015 - Tbilisi | 65/3661 | 1.78 (1.39-2.26) | 3.35 (2.04-5.51) | | <0.001 |  |
| Survey 2015 - Batumi | 21/1416 | 1.48 (0.97-2.27) | - | |  |  |
| Age between 15 to 25 years old at first anal intercourse^4^ | | | | | | |
| Survey 2010 - Tbilisi | 19/2840 | 0.67 (0.43-1.05) | 1 | | - | <0.001 |
| Survey 2012 - Tbilisi | 28/1999 | 1.40 (0.97-2.03) | 2.07 (1.15-3.70) | | 0.01 |  |
| Survey 2015 -Tbilisi | 65/2710 | 2.40 (1.88-3.06) | 3.41 (2.08-5.61) | | <0.001 |  |
| Survey 2015 -Batumi | 21/1017 | 2.06 (1.35-3.17) | - |  | |  |
| 1-Incidence Rate Ratio IRR; 2-according to the first method (17 years old as AFAI); 3- according to the second method (based on their age cohorts age between 16 to 35 years old assigned as their AFAI); 4-according to the third method (Age between 15 to 25 years old were assigned randomly as their AFAI). | | | | | | |
